# Remotely induced electrical modulation of deep brain circuits in non-human primates

**DOI:** 10.3389/fnhum.2024.1432368

**Published:** 2024-12-18

**Authors:** Carter Lybbert, Taylor Webb, Matthew G. Wilson, Keisuke Tsunoda, Jan Kubanek

**Affiliations:** ^1^Department of Biomedical Engineering, University of Utah, Salt Lake City, UT, United States; ^2^Department of Radiology and Imaging Sciences, University of Utah, Salt Lake City, UT, United States

**Keywords:** incisionless, noninvasive, induction, Lorentz force, ultrasound, magnetic field, neuromodulation

## Abstract

**Introduction:**

The combination of magnetic and focused ultrasonic fields generates focused electric fields at depth entirely noninvasively. This noninvasive method may find particularly important applications in targeted treatments of the deep brain circuits involved in mental and neurological disorders. Due to the novelty of this method, it is nonetheless unknown which parameters modulate neural activity effectively.

**Methods:**

We have investigated this issue by applying the combination of magnetic and focused ultrasonic fields to deep brain visual circuits in two non-human primates, quantifying the electroencephalographic gamma activity evoked in the visual cortex. We hypothesized that the pulse repetition frequency of the ultrasonic stimulation should be a key factor in modulating the responses, predicting that lower frequencies should elicit inhibitory effects and higher frequencies excitatory effects.

**Results:**

We replicated the results of a previous study, finding an inhibition of the evoked gamma responses by a strong magnetic field. This inhibition was only observed for the lowest frequency tested (5 Hz), and not for the higher frequencies (10 kHz and 50 kHz). These neuromodulatory effects were transient and no safety issues were noted.

**Discussion:**

We conclude that this new method can be used to transiently inhibit evoked neural activity in deep brain regions of primates, and that delivering the ultrasonic pulses at low pulse repetition frequencies maximizes the effect.

## Introduction

Mental and neurological disorders commonly affect circuits situated deep in the brain (Larson, 2014; Price and Drevets, 2012; Widge and Dougherty, 2015; Braun et al., 2018; Kuhn et al., 2009; Dandekar et al., 2018; Scangos et al., 2021). Neuromodulation has the potential to reset these circuits, but the need to modulate circuits at depth and in a selective manner has posed major challenges for existing modalities. Noninvasive methods, which include transcranial magnetic (George et al., 2000) and electrical stimulation (Lisanby, 2007; Caumo et al., 2012; Herrmann et al., 2013) lack the necessary spatial precision to modulate deep brain targets. Invasive methods, and particularly deep brain stimulation (Perlmutter and Mink, 2006; Mitchell et al., 2019), offer high precision, but the procedures require an invasive intervention, such as an implantation of stimulating leads. The risks and costs associated with these procedures have limited the spectrum of beneficiaries.

**Figure 1:**
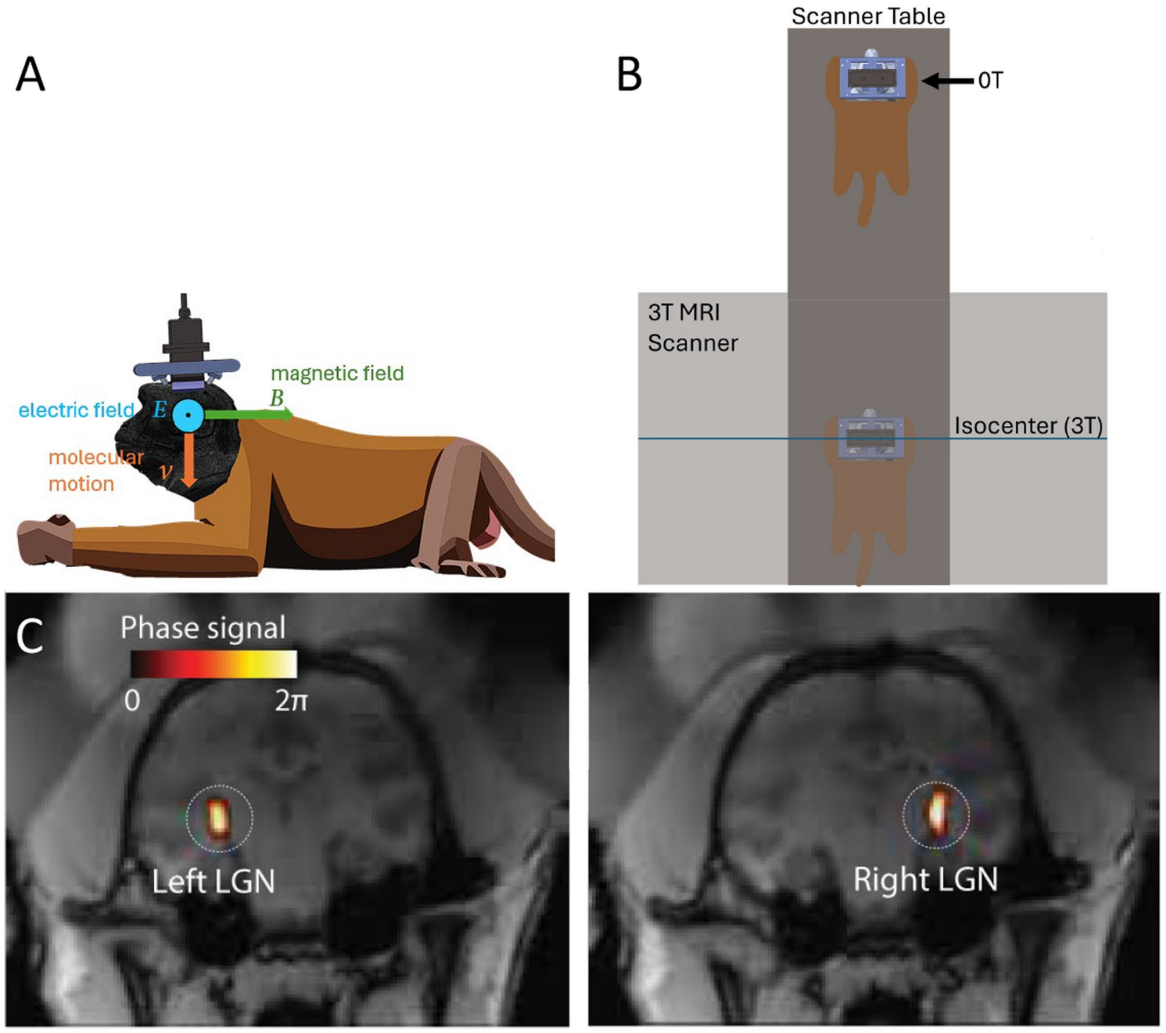
Concept and experimental setup. **(A)** Remus stimulation system and Lstim concept. An ultrasonic transducer array was attached to titanium pins mounted in the animal’s skull. This fixed positioning enabled us to deliver ultrasound into a deep brain target, the lateral geniculate nucleus (LGN) repeatably from session to session. An ultrasound wave, focused into a target with acoustic impedance Z, induces in the target motions of molecules with velocity *V*=$\frac{P}{Z}$. The pressure P (and so the velocity V) are maximal at the target. When the wave is emitted in a direction perpendicular to the magnetic field B, so that the velocity vector is perpendicular to B, the target experiences a localized electric field *E*=$\frac{\mathrm{PB}}{Z}$. **(B)** Manipulation of the magnetic field. The animal laid on an MRI table in standard sphinx position. The table allowed us to expose the animal’s brain to either high field (3T) at the isocenter of the bore and nearly zero field (0T) 3m away from the isocenter. **(C)** Validation of the ultrasound targeting. MRI thermometry was performed in each animal to validate targeting of the LGN. The details of this validation with these animals are published (Webb et al., 2022; Webb T. D. et al., 2023).

To address this issue, we have recently applied to the brain a new electrical stimulation approach that is entirely noninvasive (Webb T. et al., 2023). The approach can provide the precision of a deep brain stimulation implant while circumventing the need for a surgical procedure. To achieve that, the method combines two non-invasive forms of energies: magnetic and focused ultrasonic fields (Webb T. et al., 2023). At its focus, ultrasound displaces charged ions and molecules. The displacements are tiny and safe, but have a high velocity. A charged particle moving with high velocity perpendicularly to a strong magnetic field experiences substantial Lorentz forces (Webb T. et al., 2023). This results in induced electric intensity (Webb T. et al., 2023) that is strongest at the ultrasound focus (Figure 1). We have referred to this effect as Lstim given the Lorentz force nature of the effect (Webb T. et al., 2023). This concept has been derived theoretically (Edrich and Zhang, 1993; Norton, 2003; Kishawi and Norton, 2010; Yuan et al., 2016), and there is proof-of-concept evidence of its applicability to targeted modulation of superficial (Wang et al., 2019) and deep (Webb T. et al., 2023) brain circuits.

Nonetheless, due to the novelty of the approach—in particular with respect to applications to neural circuits—it is unknown which stimulation parameters maximize the neuromodulatory effects. Since MRI scanners readily provide a strong magnetic field, the magnetic component of the approach is fixed. The generated waveforms are therefore dictated by the pulsing scheme of the ultrasound. Previously, we have pulsed the ultrasound at a single frequency, chosen to be 200 Hz (Webb T. et al., 2023). We found that the presence of the magnetic field led to a relative decrease in evoked gamma activity. In this study, we evaluated the Lstim and neuromodulatory effects of much higher (10 kHz, 50 kHz) and much lower (5 Hz) pulse repetition frequencies (PRFs). By default, ultrasound uses relatively high carrier frequencies (e.g., 480 kHz used here and in Webb T. et al., 2023), evoking corresponding high-frequency electrical stimulation. Such high-frequency protocols generally inhibit neural activity in cases of electrical stimulation (Kilgore and Bhadra, 2014). We hypothesized that stimulation with very high PRFs would induce low frequency components, which may be more effective at stimulating neural tissue than very high frequency components (Zhang et al., 2021). Specifically, envelope modulation amounts to signal multiplication in the time domain. This is analogous to convolution in the frequency domain. If the modulation in the time domain involves a signal with high frequency components, the convolution results in a signal that contains low frequency components (Blackledge, 2005). On the other hand, modulation at a low frequency (5 Hz) is too small to broaden the spectrum, and should result in neural inhibition, similar to continuous pulses (0 Hz modulation; Webb T. et al., 2023).

We evaluated these effects in two non-human primates, using the same approach and readout as in the previous study (Webb T. et al., 2023).

## Methods

### Animals

Two adult male rhesus non-human primates (*Macaca mulatta*) participated in the experiment, subjects C and H, both 10 years old and weight 14.3 and 11.5 kg, respectively. All procedures complied with an approved Institutional Animal Care and Use Committee protocol of the University of Utah. The animals were anesthetized with isoflurane (1.0–1.25% + 1–2 L/min medical grade *O*_2_) for the duration of each session of the experiment. Throughout each experiment session vital signs were closely monitored by veterinary staff. The animals were monitored closely for 24 h after the procedure by a clinical veterinarian for any tissue damage, changes in behavior, vomiting, or food consumption, and other variables. The veterinarian provided a comprehensive written assessment following each procedure. The veterinarian noted no adverse events or effects. The ultrasonic stimuli used in this study were within the FDA 510(k) Track 3 recommendation of an ISPTA of up to 720 mW/cm^2^ for diagnostic ultrasound (FDA, 2023).

### Magnetic field

The ultrasonic stimulation was performed for half the sessions inside a Siemens Vida 3T MRI scanner (3T condition), and half the sessions outside of the MRI scanner (0T condition), for comparison of the impact of the magnetic field on the stimulation. The static magnetic field inside the bore of the scanner is relatively uniform and is directional from the front of the scanner to the rear (Van Nierop et al., 2012). The magnetic field pointed in direction perpendicular to the direction of ultrasonic stimulation (Figure 1A). The animals were positioned for all stimulations in standard sphinx position, laying on their stomach. For stimulations conducted inside the MRI, the head of the animal was placed at isocenter of the scanner. For stimulations conducted outside of the scanner, the animal was moved as far outside of the MRI scanner as possible while still on the scanner table (Figure 1B). This was 3.0 m from isocenter of the magnet, and 1.3 m from the edge of the scanner.

### Transducer setup

Ultrasonic stimulations were delivered using the Remus system (Webb et al., 2022). Briefly, a 256-element, MRI-compatible phased array transducer is inserted into a frame that is mounted onto 4 titanium pins attached to the animal’s skull. This mounting system has been previously validated to produce reproducible targeting with this particular transducer setup (Webb et al., 2022; Webb T. D. et al., 2023). The transducer is coupled to the head of the animal via a cryogel in combination with standard ultrasound gel. The coupling quality is validated prior to each session using an ultrasound imaging sequence, also described previously (Webb et al., 2022). The ultrasound was delivered into two deep brain targets, the left and right lateral geniculate nucleus (LGN) of both brain hemispheres. Targeting of the LGN was validated using MRI thermometry done previously with these animals and precisely the same transducer setup (Webb et al., 2022; Webb T. D. et al., 2023).

### Stimulation parameters and sessions

Ultrasound stimuli [480 kHz carrier frequency, 1.93 MPa amplitude *in situ* (Webb et al., 2022)] were applied to each LGN every 4 s in a strictly alternating manner (left LGN, right LGN, etc., every 4 s). Stimulation parameters were chosen such that several PRFs were assessed which were both lower than and much higher than the 200 Hz PRF that had been tested by our group previously (Webb T. et al., 2023), and such that the time-average spatial peak intensity at target (ISPTA) were all matched to 720 mW/cm^2^. We hypothesized that pulsing (i.e., modulating the envelope of) the ultrasound at very high PRFs (50 kHz) should produce low frequencies, which may have distinct neuromodulation effects from the high-frequency ultrasound carrier frequency.

The stimuli were pulsed at either 5 Hz, 10 kHz or 50 kHz PRF, with 5 Hz stimuli delivered at 5% duty cycle and 10 kHz and 50 kHz delivered each at 50% duty cycle. All were delivered at a peak pressure of 1.93 MPa. Stimulations delivered at 10 kHz or 50 kHz PRF were delivered with 100 ms pulse duration, while 5 Hz sonications were delivered with 1,000 ms pulse duration each, to match the total on-time of the other PRF sonications. Thus, each sonication was delivered with 50 ms of on-time. Stimulation was conducted for animals either fully positioned inside a 3T MRI scanner (Siemens VIDA) or translated such that the head was about 1.3 m outside of the entry plane to the bore. The magnetic field at that distance was approximately 0.02T. We referred to this level as 0T in this paper given the relatively high 3T reference. The stimuli used in this study were safely within the FDA 510(k) indices for ISPTA and ISPPA (FDA, 2023). Furthermore, the induced electric fields were safely below the recommended charge density limit of 30 *μ*C/cm^2^ (Cogan et al., 2016).

Stimuli were delivered at either 5 Hz PRF, 10 kHz PRF or 50 kHz PRF for each individual session. The individual stimuli comprised a comparable number of sessions (9 for 5 Hz, 13 for 10 kHz, and 10 for 50 kHz) to ensure that statistical results were not due to differences in power. Specifically, in subject C, 4 sessions were collected at 5 Hz, 5 were collected at 10 kHz and 5 were collected at 50 kHz. For subject H, 5 sessions were collected at 5 Hz PRF, 9 sessions were collected at 10 kHz and 5 were collected at 50 kHz. In the first session for each animal, the stimulation was first performed inside the MRI and then outside. In the second session, this order was reversed, and alternated for each subsequent stimulation session. There was at least a 2-min interval between the inside and outside the bore stimulations. No other variables other than the magnetic field were manipulated. The level of anesthesia, position of the animal, stimulation setup, electroencephalographic (EEG) monitoring, etc. were all delivered identically inside and outside the scanner. Only the MRI table was moved. Further, whether the stimulation started inside or outside the scanner was strictly alternated for each condition.

The number of stimuli delivered within each session was 80 for 5 Hz, 20 for 10 kHz and 10 for 50 kHz. These stimuli numbers were chosen as a result of technical limitations in the software, which could only provide a limited number of stimuli for sessions with higher PRFs. More specifically, for the first 8 sessions of 5 Hz insonations, we collected 80 trials per session. For the final session, 40 trials were collected. For all 13 sessions at the 10 kHz PRF, 20 stimulation trials were collected. For the first 8 sessions at 50 kHz, 10 stimulation trials were collected. In the final 2 sessions, the trial number was increased to 20 per session.

### Electrophysiological recording and analysis

To obtain high-fidelity EEG recordings of the evoked responses, we implanted four titanium pins into the skulls of the animals. The two rear pins were over the visual cortex of each monkey approximately in the P3/P4 positions in the 10/20 EEG system. The right/left pin sampled activity of the right/left visual cortex, respectively. The two frontal pins served as ground and reference. An RHS2000, Intan EEG acquisition system was used, which sampled the signals at 20 kHz and low-pass filtered the signal at 7.5 kHz. The average impedance of the EEG recording was 0.30 kOhm.

The EEG recording and the quantification of gamma activity were analogous to a previous study (Webb T. D. et al., 2023). The EEG signals were first averaged over the two channels. Samples with absolute activity greater than 300 mV were considered spurious and were removed from the analysis.

Gamma activity was assessed by a Short-Time Fourier Transform using a Hamming window method, computed with 200 ms windows, overlapping by 50%. The gamma band considered included 30 to 80 Hz activity, with a notch filter applied at 60 Hz. The gamma activity was normalized by the average gamma activity within a 1 s window preceding each stimulus, which provided a baseline for the assessment of the ultrasound and Lstim-evoked changes. Specifically, the percent change of gamma power was calculated relative to this baseline for each trial for the 4 s following stimulation. For each stimulation session, the percent change of EEG gamma power relative to the 1 s baseline was averaged across all trials in that session. This normalized the varying numbers of trials across sessions. Points with a percent change gamma value relative to the baseline of greater than 5,000% were considered spurious and were excluded from the analysis. The mean EEG gamma power percent change relative to baseline was then calculated across all sessions in each stimulation condition. The standard error of the mean was calculated for each time point. A 30 s baseline and 30 s post-period were recorded before and after the stimulation blocks of each session to monitor longer term changes.

### Statistical analysis

Throughout this article, we used non-parametric tests as the data was not necessarily normally distributed, as determined by an Anderson-Darling test. Once gamma power percent change values were calculated relative to the 1 s baseline for each session, the median percent change gamma power after ultrasonic stimulation was calculated for each session. The period of analysis post-stimulation began 200 ms after sonication offset in all cases to avoid inclusion of the electrical artifact associated with the activity of the transducer when it was on. The period of analysis extended from 200 ms post-sonication to 4 s after the onset of sonication, which coincided with the onset of the next sonication. A Wilcoxon rank-sum test was performed on the median percent change gamma power of each stimulation condition to calculate statistical significance of the difference between stimuli performed inside vs. outside of the MRI scanner, with a post-hoc Bonferroni correction for multiple comparisons. A Wilcoxon signed-rank test was used to determine if the evoked potentials were significantly different from baseline (zero), with an identical post-hoc correction. Effect sizes were calculated using Cliff’s delta and robust Cohen’s D, respectively.

## Results

We have previously found that focused ultrasound delivered to a deep brain visual region—the LGN—in non-human primates modulates gamma responses recorded over the visual cortex (Webb T. D. et al., 2023; Webb T. et al., 2023). We harnessed these findings and this approach to investigate the effects of Lstim (Webb T. et al., 2023), in which the focused ultrasound is combined with a strong magnetic field to induce a localized electric field at the target (Figure 1).

**Figure 2:**
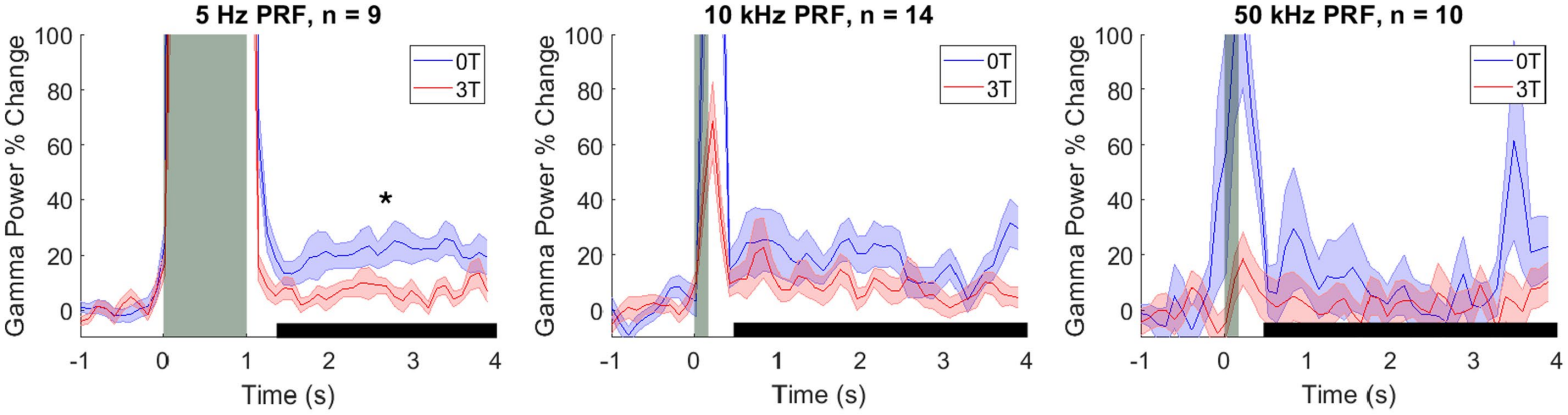
Modulation of the deep brain target by ultrasound and Lstim. Mean ± s.e.m. gamma power change relative to the 1 s baseline before stimulus onset. Shaded error bars represent the standard error of the mean across sessions at each individual time point. The vertical gray bars represent the time of the ultrasonic stimulation (1,000 ms for 5 Hz PRF and 100 ms for 10 and 50 kHz PRF). The horizontal black bars represent the analysis window. *: *p* < 0.05, two-sided Wilcoxon rank-sum test with a Bonferroni correction.

We have replicated the results of the two previous studies (Webb T. D. et al., 2023; Webb T. et al., 2023), finding that ultrasound focused into the LGN evoked a robust increase in gamma activity (Figure 2). We have quantified these effects in a time window (black horizontal bar in Figure 2) from 200 ms following the ultrasound offset (to bypass any potential stimulus-evoked artifact) to 4.0 s (which coincided with the next ultrasound pulse). In this time window, there was a median (± 25th and 75th percentile) increase of gamma by 10.1% (4.2–21.1), 8.3% (5.7–14.0), and 0.2% (−9.3–6.1), for the PRFs of 5 Hz, 10 kHz, and 50 kHz, respectively. These effects were significantly different from zero (the baseline) for 5 Hz and 10 kHz (*p* = 7.00 × 10^*−*4^; *p* = 1.27 × 10^*−*5^; two-tailed Wilcoxon signed-rank tests with Bonferroni post-hoc correction). The effect was not significant for 50 kHz (*p* = 1.00). There was a large effect size for both conditions (robust Cohen’s D = 1.15 and 1.06, respectively).

**Figure 3:**
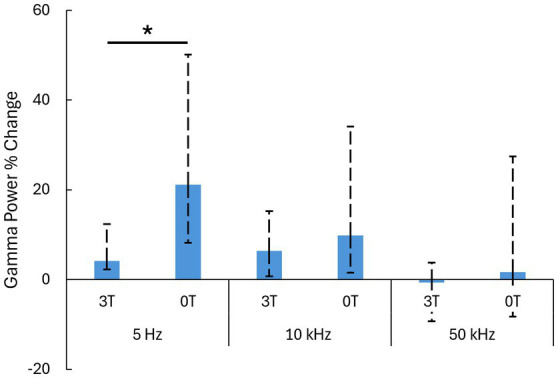
Summary of the results. Median ± 25-75th percentiles of the data (Figure 2) in the analysis window. We used these nonparametric representations and tests as the data were not necessarily normally distributed.*: *p* < 0.05; two-sided Wilcoxon rank-sum test with Bonferroni correction.

Critically, we investigated how an added magnetic field modulates these ultrasound-evoked responses. We quantified these effects for the individual PRFs. Indeed, a 3T magnetic field strongly modulated the responses (Figure 2). We reproduced the findings of our previous study (Webb T. et al., 2023), which found that low PRFs suppress the evoked gamma activity. The effect was even more pronounced in this study (Figure 2, left panel). In the magnetic field, the ultrasound-evoked response for 5 Hz PRF stimulations decreased from the initial 21.1% (12.9–29.0) to 4.2% (1.9–8.3) (median ± 25-75th percentiles). The difference was significant (*p* = 0.004, two-tailed Wilcoxon rank-sum test). The effect size was large, with a Cliff’s delta of 0.85. At 10 kHz PRF (Figure 2, middle panel) the evoked response decreased from the initial 9.8% (8.3–24.3) to 6.4% (5.7–8.9) (median ± 25-75th percentiles). The difference between the 0T and 3T conditions was not significant in this case (*p* = 0.281). There was also no significant difference between the 0T and 3T conditions at 50 kHz PRF (*p* = 1.00), 1.6% (−9.9–25.8) at 0T to −0.7 (−8.6–4.5) at 3T (median *±* 25-75th percentiles). These results are summarized in Figure 3.

We also analyzed the 30 s following each stimulation session to monitor longer-term changes in gamma power relative to the baseline gamma level. We found no significant change in gamma power at 5 Hz in either 3T or 0T conditions (*p* = 0.573, 3T; *p* = 0.652, 0T), 10 kHz (*p* = 0.497, 3T; *p* =0.588, 0T) or 50 kHz (*p* = 0.131, 3T; *p* = 0.492, 0T) as determined by a Wilcoxon rank-sum test with a Bonferroni correction.

**Figure 4:**
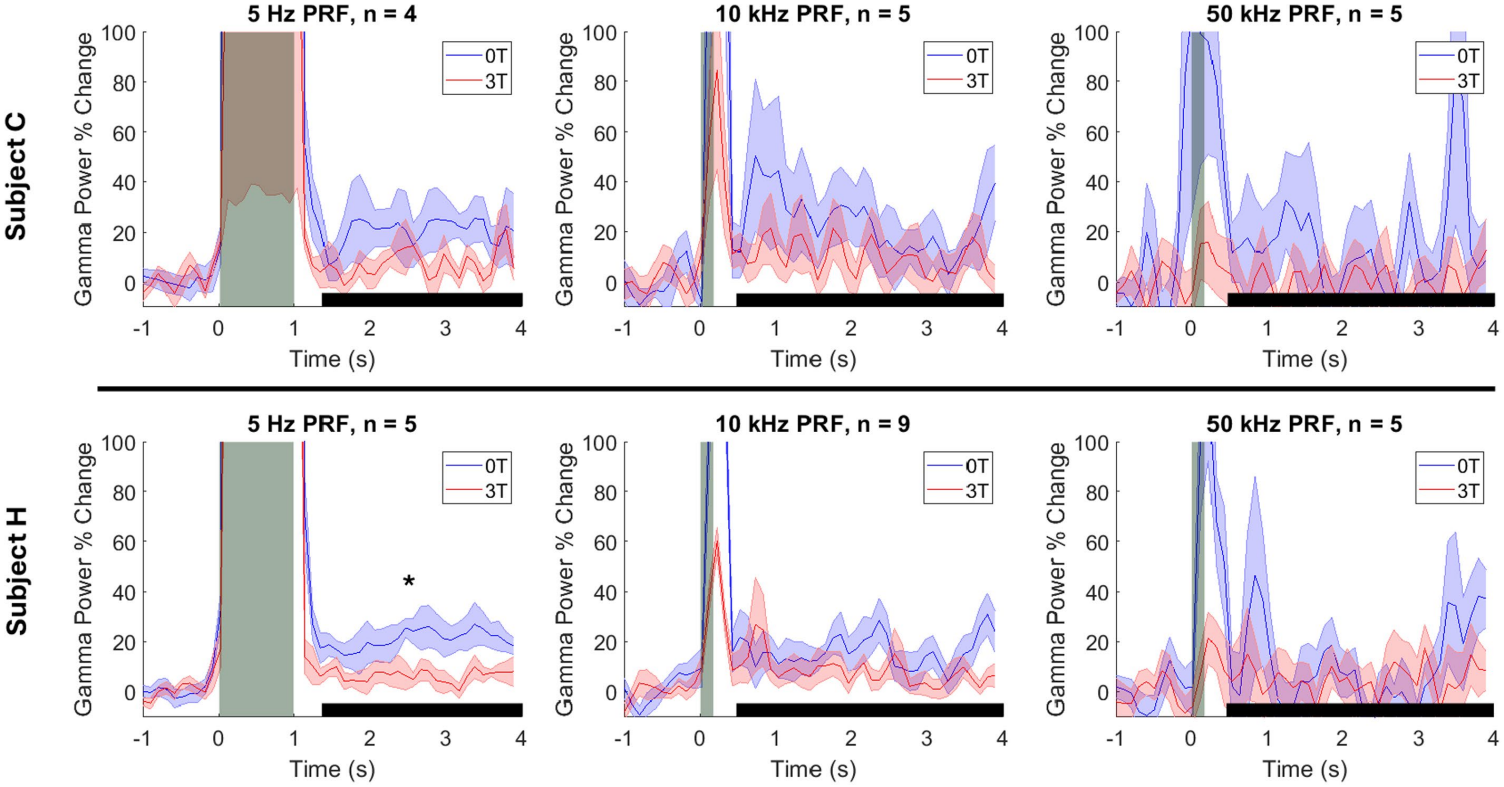
Modulation of the deep brain target by ultrasound and Lstim, separated by animal. Mean ± s.e.m. gamma power change relative to the 1 s baseline before stimulus onset. Shaded error bars represent the standard error of the mean across sessions at each individual time point. The vertical gray bars represent the time of the ultrasonic stimulation (1,000 ms for 5 Hz PRF and 100 ms for 10 and 50 kHz PRF). The horizontal black bars represent the analysis window. *: *p* < 0.05, two-sided Wilcoxon rank-sum test with a Bonferroni correction.

The effect of the magnetic field on gamma activity followed a similar trend for both animals. For subject C, the gamma changes at 5 Hz PRF were 4.2% at 3T and 21.1% at 0T (*p* = 0.343). At 10 kHz PRF, gamma changes were 5.8% at 3T and 9.2% at 0T (p = 1.00) For 50 kHz PRF, gamma changes were −1.3% at 3T and 3.8% at 0T (p = 1.00). For subject H, the gamma changes at 5 Hz were 4.7% at 3T and 19.3% at 0T (*p* = 0.048). At 10 kHz PRF, gamma changes were 5.7% at 3T and 9.3% at 0T (*p* = 0.120). For 50 kHz PRF, gamma changes were 3.4% at 3T and −0.8% at 0T (*p* = 1.00). These are summarized in Figure 4.

No detrimental effects were observed during or after the stimulation. The animals showed normal behavior following the procedures.

## Discussion

This article confirms and expands on previous findings that the combination of perpendicularly applied ultrasonic and magnetic fields modulates neural activity of deep brain regions in non-human primates. The effect, Lstim, inhibited evoked responses at a low PRF and caused no net effect at high PRFs.

The high-frequency (480 kHz) ultrasonic carrier component of Lstim used in this study induced high-frequency electric fields of the same frequency (Webb T. et al., 2023). High-frequency continuous electrical waveforms have been used for neural inhibition or conduction block (Grossman et al., 2017; Kilgore and Bhadra, 2014). This explains the effects observed in this study and in the previous study (Webb T. et al., 2023), which found an inhibition by the magnetic field of evoked neural responses for continuous or low frequency (≤ 200 Hz) PRFs. In this study, we hypothesized that modulation of this high-frequency carrier by a high-frequency rectangular envelope broadens the spectrum of frequencies (Serov, 2017), which may result in neural excitation via Lstim. Surprisingly, we found a non-statistically significant inhibitory effect even for the relatively high PRF of 10 kHz. At 50 kHz PRF we found no significant modulation. The 50 kHz data suggest that the effect may begin to switch polarity at around 50 kHz PRF. We chose the frequency of 50 kHz as the upper limit as our ultrasonic equipment was not capable of modulating the carrier envelope at higher frequencies. PRFs higher than 50 kHz should be tested in future studies using transducers with a broad bandwidth. It is possible that this frequency presents a threshold, and that even higher frequencies would produce an excitation.

This study shows that ultrasound alone can be used to excite deep brain neural circuits, finding reliable increases in gamma activity. In contrast, Lstim, which elicits high-frequency electrical stimulation through an added magnetic field, has been found to inhibit neural stimulation. Therefore, the presence or absence of the magnetic field may be able to facilitate inhibitory neuromodulatory effects. Both excitatory and inhibitory effects have important uses in applied and basic neuroscience studies. The magnetic field alone could modulate the electrophysiological responses presented in this experiment (Oliviero et al., 2011). Nonetheless, the dependency of the effect on PRF argues for an interaction between the magnetic field and the ultrasound. However, these data cannot rule out the possibility that the magnetic field causes a general decrease in neuronal excitability which dampens the effect of the ultrasonic stimulation. Further experiments strictly comparing the effects of ultrasound propagating parallel and perpendicular to the magnetic field will be needed to disentangle these effects.

The data of this study also have implications to ultrasonic neuromodulation, i.e., the application of ultrasound to the brain without a superimposed magnetic field. In this regard, we have replicated the findings of a previous study (Webb T. et al., 2023) that found that brief pulses of ultrasound applied to the LGN increase the gamma activity recorded over the main projection area of the LGN: the visual cortex. These two studies now confirm that ultrasonic modulation of deep brain neural circuits evokes gamma activity, which is a surrogate of multi-unit discharge activity (Nir et al., 2007; Manning et al., 2009). Notably, the approach taken in these studies, i.e., recording from a connected region (the visual cortex) and not directly from the targeted region (the LGN), bypasses the issue of artifacts caused by a recording electrode being impacted by the focused ultrasonic field. The modulation of the PRF in this study also has relevance to ultrasonic stimulation: relatively low PRFs (5 Hz) were more effective than high PRFs (10 kHz and 50 kHz) in evoking gamma activity. This is in line with previous suggestions (Kubanek et al., 2018; Naor et al., 2016; Tyler et al., 2018; Blackmore et al., 2019), but adds even higher PRFs to the tested repertoire.

We controlled for potential artifacts associated with the ultrasound delivery (Guo et al., 2018; Sato et al., 2018) in four ways. First, the animals were deeply anesthetized. Deep anesthesia eliminates movement in response to potential artifactual effects such as sounds (Guo et al., 2018; Sato et al., 2018). Second, we quantified the neural responses following the ultrasound offset. This way, we bypassed the potential artifacts that could be associated with the ultrasound delivery, such as electrical or electromagnetic effects. Third, the finding of no effect at 50 kHz provides a negative control. Finally, we expect the same ultrasound artifacts during measurements performed inside and outside of the MRI bore. Thus, the comparison between measurements with and without a magnetic field controls for generic artifacts caused by the ultrasound stimuli.

This article provides evidence that ultrasonic stimulation applied inside a strong magnetic field, such as inside an MRI scanner, invokes the Lorentz force effect, which, as this article shows, can have strong neuromodulatory consequences. The Lstim effects are maximized when the vectors of the magnetic field and of the ultrasonic beam are perpendicular. The presence of this previously underappreciated effect should be taken into account any time ultrasound is applied inside an MRI scanner.

Compared with other neuromodulatory approaches, Lstim has some advantages. Traditional electrical stimulation targeted to specific brain circuits relies on implanting electrodes into the desired stimulation location (van Balken et al., 2004; Larson, 2014; Lempka and Patil, 2018). While this approach produces spatially targeted electric fields, it requires direct implantation of electrodes into neural tissue, which limits the safety and longevity of the implant. Further, once implantation is completed, stimulation can only be delivered at the implanted location, resulting in limited spatial flexibility. The Lstim approach described in this experiment allows for focal electrical stimulation of deep brain circuits without invasive electrode implantation and the associated risks. It also allows for low-latency steering of the stimulation location, allowing for flexible stimulation of multiple brain targets in a single experiment. Other non-invasive approaches to brain stimulation suffer from either poor spatial resolution or poor depth penetration into deep brain regions, or both. For example, transcranial magnetic stimulation can induce electric currents in the cortex, but not deeper (Zibman et al., 2021). Transcranial direct or alternating current stimulation produces limited intensity in deep brain circuits and only attains several centimeters spatial precision in the brain (Caumo et al., 2012), even when applied with spatially interfering fields (Grossman et al., 2017). In comparison, Lstim allows for electrical stimulation of deep brain circuits with spatial specificity of several millimeters or smaller (Webb T. D. et al., 2023; Menz et al., 2013), given its tight bound to the spatial location of the applied ultrasound stimuli.

This study has certain caveats. First, the monkeys were anesthetized in this study. This was critical to avoid movement and enable exposure of the animals to the strong magnetic field of an MRI machine. Anesthesia is known to influence neural responses to neuromodulation (Ye et al., 2016; Lee et al., 2018), which could impact the results and induce a net neuroinhibitory bias. Future studies should attempt to create strong magnetic fields while a research subject is awake. Given that we did not detect detrimental effects, the effects of Lstim could conceivably also be evaluated in human subjects inside an MRI scanner. In fact, ultrasound has been applied to the human brain inside strong magnetic fields, up to 7T, and no detrimental effects were reported (Lee et al., 2016; Ai et al., 2018). Second, we have evaluated the effects using EEG. Although the remote readout was beneficial to avoid artifacts of the ultrasound impacting a recording electrode, this readout is indirect. A behavioral readout (e.g., Webb et al., 2022; Webb T. D. et al., 2023) could provide a more direct and relevant link to the activity modulated at the target. A remote optical recording method could also be considered. Finally, we have designed this study to systematically evaluate the effect of PRF, quantifying transient changes to gamma activity following each stimulus. The study was not designed to systematically evaluate any durable effects that could be attributed to Lstim. Although transient effects are useful for diagnostic or guidance applications, applications that require a sustained reset of malfunctioning circuits will rest on durable effects. Future studies should be designed to elicit and investigate such effects.

Lstim provides an entirely non-invasive way to modulate deep brain regions in humans. The neuromodulatory effects could be used for systematic and flexible investigations of brain function in primates and humans. By systematically perturbing individual brain targets using Lstim, clinicians and researchers could be empowered to determine which targets are causally involved in given disease signs, symptoms, or behaviors. This could further the causal understanding of the neural substrates of neurological and mental disorders and of basic brain function in humans.

In summary, this study combined strong magnetic and focused ultrasonic fields to modulate deep brain regions of non-human primates. The elicited Lorentz force effects are found to modulate evoked neural activity. The effects point in the inhibitory direction for low-medium range of pulse repetition frequencies. The neuromodulatory effects associated with this noninvasive targeted approach could be harnessed to advance the causal understanding of the neural substrates of normal function and dysfunction.

## Data Availability

The raw data supporting the conclusions of this article will be made available by the authors, without undue reservation.
